# Evaluation of water‐assisted UV‐C light and its additive effect with peracetic acid for the inactivation of *Listeria monocytogenes*, *Salmonella enterica* and murine norovirus on whole and fresh‐cut strawberries during shelf‐life

**DOI:** 10.1002/jsfa.11913

**Published:** 2022-05-10

**Authors:** Jordi Ortiz‐Solà, Antonio Valero, Maribel Abadias, Iolanda Nicolau‐Lapeña, Inmaculada Viñas

**Affiliations:** ^1^ Universitat de Lleida, Food Technology Department Agrotecnio‐Cerca Center, 25001 Lleida Carrer de Jaume II, 69 Spain; ^2^ Universidad de Córdoba Faculty of Veterinary Medicine, Department of Food science and Technology, 14014 Córdoba Avenida de Medina Azahara, 1 Spain; ^3^ IRTA, Postharvest Programme, Edifici Fruitcentre, Parc Científic i Tecnològic Agroalimentari de Lleida, 25003 Lleida Parc de Gardeny Spain

**Keywords:** sanitization, cold storage, pathogen reduction, fruit, chlorine

## Abstract

**BACKGROUND:**

The purpose of the present study was to examine the inactivation of *Salmonella enterica* (50 μL; 10^9^ CFU g^−1^)*, Listeria monocytogenes* (50 μL; 10^9^ CFU g^−1^)*,* and murine norovirus (MNV‐1; 50 μL; 10^7^ 50% tissue culture infectious dose (TCID_50_) mL^−1^) on whole and fresh‐cut strawberries after 2 min disinfection treatments (water (H_2_O), chlorine 200 mg L^−1^ (NaClO), water‐assisted ultraviolet‐C (UV‐C) (WUV), and the combination WUV and 40 mg L^−1^ of PA (WUV + PA)) in a water tank (15 L) equipped with 4 UV‐C lamps (17.2 W each), and after 7 days of cold storage (4 and 10 °C). For MNV‐1, dry UV‐C treatment (DUV) was also tested. For all UV‐C treatments, an irradiation dose of 1.3 kJ m^−2^ was used.

**RESULTS:**

When strawberries were washed with WUV, *L. monocytogenes* and *S. enterica* were reduced by 2.8 and 2.2 log CFU g^−1^, respectively. The addition of 40 mg L^−1^ of PA to WUV (WUV + PA) increased the reduction range of *L. monocytogenes* and *S. enterica* by 1.9 and 0.8 log, respectively. Regarding the wash water, no pathogens were recovered after the WUV + PA treatment (detection limit 50 CFU mL^−1^). Depending on storage conditions (7 days at 4 or 10 °C), reductions observed were 0.5 to 2.0 log for *S. enterica* and 0.5 to 3.0 log for *L. monocytogenes*. The reductions in MNV‐1 titer after disinfection treatments ranged from 1.3 to 1.7 log. No significant differences between storage conditions were observed for MNV‐1: titers did not decline or were reduced up to 0.3 log after 7 days of cold storage.

**CONCLUSION:**

The three‐way action for disinfecting strawberries by UV‐C irradiation and PA, plus the physical removal of the microorganisms by agitated water, are effective against foodborne pathogens on strawberries and water wash. During storage, WUV had a larger impact on the inactivation kinetics of *S. enterica*. Storage had little impact on MNV‐1 inactivation. © 2022 The Authors. *Journal of The Science of Food and Agriculture* published by John Wiley & Sons Ltd on behalf of Society of Chemical Industry.

## Introduction

Demand for fresh strawberries is increasing because they combine a delicious flavour with a wide range of nutrients and bioactive phytochemicals that are believed to be beneficial for human health.^1^ However, due to their high water content (up to 90%), soft texture, and high level of respiration, strawberries are very exposed to microbial contamination, and are thus very perishable.^2^ In fact, the shelf‐life of fresh strawberries is very short (1–2 days) at room temperature, and chilling temperatures (<7 °C) are needed to keep them fresh for long time (7–8 days).^3^ Moreover, strawberries have been linked to safety issues associated with foodborne pathogens, such as bacteria (*Salmonella* spp. and *Listeria monocytogenes*), viruses (norovirus and hepatitis A) and zoonotic parasites.^4^, ^5^, ^6^ Survival of *Salmonella* spp. and *L. monocytogenes* has been reported on whole and fresh‐cut strawberries, surviving at different temperatures (4, 5, 10, 15, 22, and 24 °C) during the shelf life of the fresh product (ca. 7 days).^7^, ^8^, ^9^ Regarding enteric viruses, previous investigations reported that human norovirus and adenovirus decreased rapidly on strawberries stored at room temperature (21 °C), with TFL‐values (time for the first log‐unit reduction) of only 1 day (95% CI of 0.6–1 and 0.8–1 days, respectively). On the other hand, no decrease was observed for murine norovirus on strawberries at refrigeration temperatures.^10^

To prevent microbial prevalence or growth, disinfection methods and preservation procedures are included in the fresh produce.^11^ Currently, sodium hypochlorite is the most commonly used disinfectant in the industry to reduce the microbial load of fresh and frozen produce. However, chlorine has a limted action, especially for MNV‐1 inactivation, being maximum reductions in fresh produce lower than 1.2 log units.^12^ Moreover, a growing concern about its by‐products, which are harmful to consumers and environment, has led producers to search for alternative sanitation approaches.^13^ Among them, short‐wave ultraviolet light (ultraviolet‐C, UV‐C) has been proposed for its inexpensiveness and microbicidal effect on foodborne pathogens. Ultraviolet‐C has a direct harmful action on pathogen DNA assembly leading to inactivation and cell death, without producing harmful side products for the consumers.^14^ Thus, UV‐based procedures are being applied for the decontamination of food, food‐contact surfaces, and water washing.^15^ Unfortunately, this technology has its own disadvantages such as product‐overheating and a shadowing effect.^16^ For this reason, water‐assisted alternatives to disinfection with UV‐C light (WUV) enhance the accessibility of UV‐C and improve the removal of microorganisms from rough and hidden surfaces,^17^, ^18^ while reducing the probability of fruit overheating in comparison with air‐transmitted UV‐C.^19^, ^20^ The germicidal effect of UV‐C light would also act in water, reducing the surviving microorganisms, which could have been released from the fruits in this washing step, preventing cross‐contaminations in following work‐flow cycles.^21^, ^22^ Moreover, the addition of a chemical sanitizer to WUV treatments could enhance the germicidal effects.^22^ For instance, previous studies have evaluated peracetic acid, an oxidizing‐sanitizer that an appropriate alternative to chlorine solutions due to its robustness against suspended organic matter, changes in pH and temperature, and the non‐toxicity of its disinfection by‐products (water, oxygen, and acetic acid).^23^ Our research group has previously studied the efficacy of a water‐assisted UV‐C technology, alone or combined with PA for the reduction of natural microbiota, *L. innocua* and *S. enterica* in fresh produce, including strawberries.^15^, ^18^, ^19^ In this regard, the study of the use of PA at different concentrations (20, 40 and 80 mg L^−1^) revealed that 40 mg L^−1^ were needed to decrease *L. innocua* populations significantly in strawberries (ca. 4 log units).^24^ In another study,^25^ the use of a WUV system for up to 5 min significantly reduced artificially inoculated populations (ca. 4 log units for *L. innocua* and ca. 3 log units for *S. enterica*) in strawberries. In the same study, the incorporation of PA at 40 or 80 mg L^−1^ increased the sanitizing effect, especially in the washing water, in which non‐detectable counts for *L. innocua* were obtained, preventing cross‐contamination and making it possible to reduce treatment time (from 5 to 2 min).^25^ The authors concluded that a treatment consisting of UV irradiation for 2 min (1.3 kJ m^−2^) in strawberries immersed in a 40 mg L^−1^ PA solution was the efficient option and whose results could be comparable to the use of chlorine at 200 mg L^−1 24^.

Several studies can be found on the search for effective and feasible methods to prolong the shelf life of strawberries.^3^, ^7^, ^8^, ^9^ However, as far as we are aware, very few studies have focused on monitoring the survival of pathogenic bacteria and viruses during the shelf life of strawberries after being submitted to sanitizing treatments. The treatments proposed in the present study could induce changes in both the fruit matrix (hormesis) and in the selected pathogens (photoreparation, sensibilization, or acid‐shock developed resistance) that could have an impact on their fate during shelf life.^26^, ^27^ The present study evaluates sanitizing strategies based on water, chemical sanitizers, and UV‐C technology to inactivate foodborne pathogenic bacteria and murine norovirus (MNV‐1) (a human norovirus surrogate) from strawberries, and to monitor populations of pathogenic microorganisms during the shelf‐life (7 days at 4 and 10 °C) of whole and fresh‐cut strawberries.

## Materials and Methods

### Plant processing

Fresh strawberries (*Fragaria × ananassa*) were purchased from local distributors in Lleida (Spain). Samples with visible physical injuries, or that were rotten, were discarded and only intact, healthy and ripe fruits with similar size and weight (approximately 25 g) were selected. Before the experiment, the peduncles and leaves of the fruits were carefully discarded. The fruits were kept in trays overnight at 4 °C without any additional discarding or processing until they were submitted to subsequent sanitation treatments.

### Materials

All synthetic culture media, such as tryptone soy broth (TSB), tryptone soy agar (TSA), Palcam base agar, Palcam supplement, xylose‐lysine‐desoxycholate agar (XLD), yeast extract (YE), and peptone were obtained from Biokar Diagnostics (Allonne, France). Dey‐Engley broth was obtained from Fluka (Madrid, Spain). Dulbecco's modified Eagle medium (DMEM) and fetal bovine serum (FBS) were purchased from Hyclone (Pittsburgh, PA, USA); 200 mM glutamine, 10 mM *N*‐2‐hydroxyethylpiperazine‐N0‐2‐ethanesulfonic acid, and 1% penicillin–streptomycin were obtained from Biowest (Nuaillé, France).

### Microbial culture conditions and inoculum preparation

For pathogenic bacteria, a five‐strain cocktail of *Listeria monocytogenes* or *Salmonella enterica* was used as inoculum (Table 1). Cultures were prepared as described in Ortiz‐Solà *et al*. (2020).^29^ The day before the experiment, fresh strawberries were inoculated separately with 10^9^ CFU mL^−1^ of *L. monocytogenes* and *S. enterica* inoculum, by pipetting 50 μL in small droplets on the fruit surface. Once dried (1–2 h at room temperature), strawberries were stored at 4 ± 1 °C overnight.

**Table 1:** Bacterial strains used in the experiment

Specie		Collection number
*Listeria monocytogenes*	4b	CECT^a^‐4032
*L. monocytogenes*	1/2 a	Isolated in Lab^c^
*L. monocytogenes*	01 feb	CECT‐4031
*L. monocytogenes*	3a	CECT‐933
*L. monocytogenes*	4d	CECT‐940
*Salmonella enterica* subsp. *enterica*	Agona	ATCC^b^ BAA‐707
*S. enterica* subsp. *enterica*	Michigan	ATCC BAA‐709
*S. enterica* subsp. *enterica*	Montevideo	ATCC BAA‐710
*S. enterica* subsp. *enterica*	Gaminara	ATCC BAA‐711
*S. enterica* subsp. *enterica*	Enteritidis	CECT‐4300

^a^ Colección española de cultivos tipo.

^b^ American‐type culture collection.

^c^ Abadias (2008).^27^

For virus inoculum preparation, the murine norovirus (MNV‐1) was used. The MNV‐1 stocks were propagated after 2 days of murine macrophage cell line RAW 264.7 infection by three freeze–thaw cycles followed by centrifugation at 660 × g for 30 min.^30^ Cell line and MNV‐1 were kindly provided by Prof. H. W. Virgin (Washington University School of Medicine, WA, USA). Infectious viruses were enumerated by determining the 50 % tissue culture infectious dose (TCID_50_). Eight wells per dilution and 20 μL of inoculum per well were used following the indications in the Spearman–Karber method.^31^ Stocks of MNV‐1 were frozen until use (−80 °C). RAW 264.7 cells were cultured in DMEM supplemented as described in Ortiz‐Solà *et al*. (2020).^29^ The cell line was maintained at 37 ± 1 °C in a 5 % CO_2_ humidified incubator (NU‐4750, NuAire, Plymouth, MN, USA) in T75 flasks (Nunc, Thermo Fisher, Waltham, MA, USA) with 85 % of relative humidity (RH).

### UV‐C water–assisted tank equipment

For washing treatments, batches of 40 whole strawberries were immersed in agitated cold (8 ± 2 °C) tap water at a ratio of 1:12 (kg of product: L of water) using a water‐assisted laboratory scale equipment LAB‐UVC‐Gama (UV‐Consulting Peschl España, Castellón, Spain). This device is composed of a water tank (15 L capacity) equipped with 4 short‐wave ultraviolet light lamps irradiating a power of 17.2 W each (GPH303T5L/4, 254 nm), a recirculating system connected to a water pump and an aeration technology that provides bubbling.^18^ Before water UV‐C treatments, the four lamps were preheated during 10 min, to reach the maximum irradiance (10.5 W m^−2^). Before and after each treatment, temperature was measured using an infrared thermometer DualTemp Pro (Labprocess Distribuciones, Barcelona, Spain) and irradiance was measured through an orifice located in the lid of the equipment using a UV‐sensor EasyH1 (Peschl Ultraviolet, Mainz, Germany).

### Sanitation washing treatments and fruit processing

Preliminary selection of the water‐assisted ultraviolet light (WUV) and peracetic acid (PA) dose was based on previous investigations reported by our research group.^24^, ^25^ Regarding the disinfection experiments of the artificially inoculated bacteria on strawberries, four treatments were evaluated: WUV, a combination of WUV and PA at 40 mg L^−1^ (WUV + PA), tap water (H_2_O), and 200 mg L^−1^ of sodium hypochlorite (NaClO) (pH 6.5). All treatments were tested during 2 min exposure, corresponding to 1.3 kJ m^−2^ irradiation dose, in the case of WUV and WUV + PA. After NaClO disinfection, strawberries were rinsed in tap cold water (8.0 ± 0.5 °C) during 2 min. For the application of H_2_O and NaClO treatments, the same device was used but the UV‐C lamps were switched off. The water was agitated by recirculation through a water pump and by air injection (set at 100 kPa).

In the case of MNV‐1, the treatments mentioned above were carried out, except for the H_2_O treatment. Additionally, a one‐sided ‘dry’ UV‐C (DUV) treatment without water immersion was also performed. Sample fruits treated with DUV were arranged along a biosafety laminar air cabinet (class II — type A) with the inoculated‐side upwards and just below the UV‐C light following the same conditions and time exposure mentioned above with an irradiation dose of 1.3 kJ m^−2^.

For water‐assisted treatments, water parameters including pH and oxidation–reduction potential (ORP) were measured at the beginning and at the end of each treatment. The ORP and pH values were measured in a pH meter (GLP22, Crison, Alella, Spain) equipped with a pH probe (ref. 52–03, Crison) or ORP probe (ref.62–51 Hach, Vésenaz, Geneva). After washing, fruits were let to drain the excess of water at room temperature (22 °C) during 1–2 h on a metal grid in a biological safety cabinet. Once dried, three whole strawberries per treatment were stored in polypropylene (PP) trays (375 mL). The trays were sealed with tray‐lidding film by self‐sealing lab scale equipment (AK‐Ramon TS‐150, Barcelona, Spain). The film used was artificially perforated with a 100 μm diameter needle, making seven orifices homogeneously distributed per tray. In preliminary tests (data not shown), these conditions gave the best storage quality results for strawberries throughout experimental time. Samples were stored at 4 °C and 10 °C in dark conditions.

For the fresh‐cut strawberry studies, whole strawberries were washed with the same treatments as above. The strawberries were left to dry at room temperature. Once dried, they were cut in the laboratory with a sterile knife before being sealed in the PP trays. Afterwards, they were packaged and stored using the same conditions as those used for whole strawberries.

### Microbiological analysis

During the experiment, reductions of *S. enterica, L. monocytogenes* and the infectivity of MNV‐1 were monitored on day 0 (after disinfection procedures) and 7 (after storage at 4 and 10 °C). For fresh presentation, a whole strawberry was analyzed, whereas for half‐cut strawberries, two pieces (simulating a whole strawberry) were used. Each experiment was repeated once. Three biological replicates (three trays) per treatment and sampling time were used for microbiological analysis.

#### Bacterial counts

For bacterial analysis, appropriate tenfold solutions in saline peptone (SP, 8.5 g L^−1^ NaCl, 1 g L^−1^ peptone) of the homogenates were plated in duplicate on Palcam agar and XLD for the determination of *L. monocytogenes* and *S. enterica*, respectively. Palcam and XLD plates were incubated at 37 °C for 48 or 24 h, respectively. Results were calculated as colony forming units per g (CFU g^−1^) and expressed as log CFU g^−1^. The detection limit was 1.30 log CFU g^−1^ (20 CFU g^−1^). Logarithmic reductions of the pathogens obtained after washing treatments were calculated by the following equation (Eqn 1):

(1) $$\mathrm{Log}\;\text{reductions}\;\left({\mathrm{Log}}_{\mathrm{dis}}\right)=\mathrm{Log}\;{\text{(𝑁}}_{0})–\mathrm{Log}\;\left(N_{t}\right)$$

where 𝑁_0_ is the mean of the initial population of untreated strawberries, and *N*_t_ is the population obtained after washing disinfection (CFU g^−1^).

Regarding the effect of storage variables after 7 days, logarithmic reductions of the pathogens achieved were calculated by the following equation (Eqn 2):

(2) $$\mathrm{Log}\;\text{reductions}\;\left({\mathrm{Log}}_{\text{stor}}\right)=\mathrm{Log}\;{\text{(𝑁}}_{t})–\mathrm{Log}\;\left(N_{d}\right)$$

where 𝑁_t_ is the population obtained after washing procedures, and *N*_d_ is the population obtained after 7 days of storage (CFU g^−1^).

Populations of bacteria were also determined in wash water after the sanitation treatment. Duplicate wash water samples (1 mL) s were diluted directly in 9 mL Dey‐Engley (DE) (Biokar Diagnostics) plated as described previously and incubated at 37 °C for detection if no colonies were present on plates. Results were expressed as log CFU mL^−1^. When counts were below the limit of detection (50 CFU mL^−1^), and presence was confirmed by DE color change followed by confirmation in selective medium, an arbitrary value of ½ limit of detection (25 CFU mL^−1^) was assigned. If no change in DE color was observed, no presence (0 log CFU mL^−1^) was assumed.

#### MNV‐1 determination

For MNV‐1 determination, the virus was extracted from the treated samples as described in Ortiz‐Solà *et al*. (2020).^29^ Briefly, confluent RAW 264.7 cells with supplemented DMEM 10% were transferred to 96‐well microtiter plates (ThermoFisher, Waltham, MA, USA). Micro‐plates were stored at 37 ± 1 °C in a 5% CO_2_ and 85% of relative humidity (RH) during 24 ± 2 h. Afterwards, DMEM 10% was removed and 20 μL per well of the tenfold dilutions with phosphate buffered solution (PBS) of each extracted sample were inoculated into 96 wells of the microtiter plates of confluent RAW 264.7. Plates were incubated under the same conditions described above. After 1 h incubation, 150 μL per well of DMEM supplemented with 2% FBS was added and incubated for 2–3 days at 5% CO_2_ and 85% HR. Then, RAW 264.7 monolayers with cytotoxic effects were observed by visual examination using the optical inverse microscope. MNV‐ 1 positive sample was used as norovirus control. Negative controls were studied using PBS (2 M NaNO_3_, 1% beef extract, and 0.1% Triton X‐100).

The MNV‐1 infectivity of each treated strawberry was calculated by determining the TCID_50_ with eight wells per dilution and 20 μL of inoculum per well. The numbers of wells with cytopathic effect were documented. The reduction of the infectivity was calculated as log (TCID_50_ mL^−1^), using Eqns (1) and (2).^32^

### Statistical analysis

The data were analyzed with JMP PRO 14.1.0 (SAS Institute Inc., Cary, NC, USA) and verified for normal distribution and homoscedasticity of residues. To investigate the influence of variable interactions (disinfection treatments, food matrices and storage temperatures) on the total logarithmic reductions, Generalized Linear Regression models were also implemented in R v3.6.3.^33^ Both single and interaction effects were performed and the goodness of fit of the models was assessed using the root mean squared error (RMSE); Akaike information criterion (AIC) and Bayesian information criterion (BIC). All data were checked for significant differences by analysis of variance (ANOVA). When significant differences were found (*p* < 0.05) further tests were performed:   Tukey's honestly significant difference (HSD) and Student's *t*‐test. Differences were considered statistically significant if the associated probability (P) was ≤0.05.

## Results and discussion

### Properties of washing water

Water used to wash strawberries was controlled during each treatment for each pathogenic microorganism. For those treatments with no chemical solution (control H_2_O or WUV), pH values were 7.5 ± 0.2, while for those with WUV + PA (40 mg L^−1^) or NaClO (200 mg L^−1^), pH values were 5.0 ± 0.4 or 7.0 ± 0.3, respectively. For the same conditions, oxidation–reduction potential (ORP) values were 252.5 ± 11.1 (control or WUV), 509.2 ± 16.2 (WUV + PA), and 876.5 ± 4.3 (NaClO). Washing treatments were carried out at 8.1 ± 0.6 °C.

### Foodborne pathogenic bacteria inactivation on strawberries and wash water after disinfection treatments

Mean initial counts of *L. monocytogenes* and *S. enterica* on fresh strawberries were 5.8 ± 0.3 and 6.7 ± 0.3 log CFU g^−1^, respectively (data not shown). After 2 min of washing treatments, reductions obtained with WUV without chemical agents (1.3 kJ m^−2^), were 2.8 and 2.2 log_dis_ for *L. monocytogenes* and *S. enterica*, respectively, which did not improve the efficacy of water‐washing (H_2_O) without sanitizers (2.7 and 2.1 log_dis_ reduction, respectively) (Table 2). The reductions in this case, were therefore attributed to a physical detachment of the microorganisms from the strawberry surface to the water. However, the combined treatment WUV + PA effectively reduced both pathogenic bacteria on the surface of strawberries by ≥3.0 log_dis_. Therefore, in the present work, the WUV + PA treatment improved the effectiveness of WUV disinfection alone (*P* < 0.05), by 1.9 and 0.8 log_dis_ for *L. monocytogenes* and *S. enterica*, respectively. To gain insight on the potential effects and interactions between disinfection treatments and the strawberry matrix (i.e., whole and fresh‐cut) on microbial reductions, GLM models were applied. Interactions were considered as GLM models provided better goodness‐of‐fit indexes than single effects models. The observed results were confirmed by the GLM models as for *Salmonella* and *L. monocytogenes*; the only significant factor was the application of WUV + PA (*P* ≤ 0.05). Interactions between type of disinfection treatment and strawberry matrix were not significant, meaning that similar effects can be achieved in whole and fresh‐cut strawberries (Tables 3 and 4).

**Table 2:** Mean and standard deviation (SD) values (n = 3) of the log reduction (Log_dis_) of *Listeria monocytogenes* and *Salmonella enterica* as a function of the applied disinfection treatments on strawberries

Disinfection treatment^a^	Reduction (log_dis_) (mean ± SD)^b^	
	*L. monocytogenes*	*S. enterica*
H_2_O	2.72 ± 0.59^Ab^	2.11 ± 0.58^Ab^
NaClO	3.47 ± 0.97^Aab^	2.65 ± 0.19^Aab^
WUV	2.79 ± 0.79^Ab^	2.22 ± 0.21^Ab^
WUV + PA	4.64 ± 0.39^Ba^	3.04 ± 0.35^Aa^

^a^ H_2_O: control samples (washing with tap water), NaClO: hypochlorite solution (200 mg L^−1^), WUV: ultraviolet disinfection, WUV + PA: ultraviolet disinfection combined with peracetic acid (40 mg L^−1^).

^b^ Upper case letters indicate significant differences between pathogenic bacteria according to the Student *t‐*test (*P* < 0.05). Lowercase letters indicate significant differences among disinfection treatments according to the Tukey honestly significant difference (HSD) *post hoc* test (*P* < 0.05).

**Table 3:** Estimations of the generalized linear regression models for the calculated log reductions after washing and disinfection treatments (Log_dis_) of *S. enterica* in whole and fresh‐cut strawberries

Variable^**a**^	Coefficient (95%, C.I.)	S.E.	*P*‐value^b^
Intercept	*2.22 (1.66, 2.78)*	*0.26*	*<0.001*
NaClO	0.57 (−0.15, 1.30)	0.34	0.111
WUV	−0.38 (−1.10, 0.34)	0.34	0.274
*WUV + PA*	*1.00 (0.28, 1.73)*	*0.34*	*0.010*
Whole	−0.21 (−1.01, 0.58)	0.37	0.568
NaClO * Whole^c^	−0.07 (−1.09, 0.96)	0.48	0.893
WUV * Whole	0.98 (−0.04, 2.00)	0.48	0.059
WUV + PA * Whole	−0.15 (−1.18, 0.87)	0.48	0.756
**Goodness of fit statistics** ^d^	RMSE: 0.609	AIC: 26.78	BIC: 36.60

^a^ Reference categories in the models were: disinfection treatment = H_2_O (control); Matrix = fresh‐cut strawberry.

^b^ Variables considered significant (*P* < 0.05) are in italics.

^c^ Interaction terms.

^d^ RMSE, root mean squared error; AIC, Akaike information criterion; BIC, Bayesian information criterion.

**Table 4:** Estimations of the generalized linear regression models for the calculated log reductions after washing and disinfection treatments (Log_dis_) of *Listeria monocytogenes* in whole and fresh‐cut strawberries

Variable^**a**^	Coefficient (95%, C.I.)	S.E.	*P*‐value^b^
Intercept	*1.71 (0.69, 2.74)*	*0.48*	*0.003*
NaClO	1.45 (−0.00, 2.90)	0.69	0.051
WUV	0.02 (−1.43, 1.48)	0.69	0.974
*WUV + PA*	*2.83 (1.37, 4.28)*	*0.69*	*0.001*
Whole	2.00 (0.55, 3.46)	0.69	0.010
NaClO * Whole^c^	−1.40 (−3.45, 0.65)	0.97	0.168
WUV * Whole	0.09 (−1.96, 2.15)	0.97	0.925
WUV + PA * Whole	−1.80 (−3.86, 0.26)	0.97	0.082
**Goodness of fit statistics** ^d^	RMSE: 0.916	AIC: 68.00	BIC: 78.60

^a^ Reference categories in the models were: disinfection treatment = H_2_O (control); Matrix = fresh‐cut strawberry.

^b^ Variables considered significant (*P* < 0.05) are in italics.

^c^ Interaction terms.

^d^ RMSE, root mean squared error; AIC, Akaike information criterion; BIC, Bayesian information criterion.

The disinfection action with the integrated strategies involving UV‐C light and PA, plus the physical removal of the microorganisms due to the water, provided greater efficacy against foodborne pathogens than the treatments used in the experiment (H_2_O or WUV), as stated in previous work.^24^, ^25^ Moreover, although pathogens on the strawberry surface were not completely removed or inactivated, results confirmed that the effect of WUV + PA treatment was comparable (for *S. enterica* sanitation, 3.04 log_dis_) and even superior (for *L. monocytogenes* 4.64 log_dis_) to disinfection with free chlorine (NaClO; 200 mg L^−1^) (*P* > 0.05), treatment that achieved 3.47 and 2.65 log_dis_ reductions, respectively. Previous investigations suggest an additive action when using the combination of 10 mg L^−1^ of PA and UV‐C (0.1 kJ m^−2^) for the inactivation of *S. enteritidis* (6.2 log reduction) in peptone water in comparison with the single treatments (1.9 and 2.6 log reduction for PA and UV‐C, respectively).^34^, ^35^ In our study, results shown that the combined treatment WUV + PA was generally more effective against *L. monocytogenes* when compared with *S. enterica* (*P* ≤ 0.05). In general, the additive effect of integrated strategies involving UV‐C irradiation and chemicals for the decontamination of inoculated pathogens in fresh produce has been shown to depend not only on the UV‐C dose and the chemical compounds' concentrations but on the indigenous microbiota and the target microorganism studied.^36^, ^37^ This effect could be related to the different ability of pathogens to interact with the plant associated microbiota, or to internalize and attach to the plant tissue during overnight incubation, which could have reduced the accessibility of UV‐C and PA or led to induced resistance of bacteria against antimicrobial mechanisms.^38^, ^39^

Regarding the effect of the assayed technologies on the microbial populations in the process water after washing for 2 min, results showed that the average populations of *L. monocytogenes* and *S. enterica* after control tap water (H_2_O) were 4.5 ± 0.1 and 4.6 ± 0.2 log CFU mL^−1^, respectively (Table 5). This microbial concentration could be attributed to the transfer of the bacteria from fruit surface to water due to the physical action of water pressure, agitation, and aeration (bubbles), explaining the reduction of microbial load in strawberries in H_2_O control, as detailed above. For WUV treatment alone, the levels of *L. monocytogenes* and *S. enterica* were significantly higher than the washing treatments with chemical agents (0.9 ± 0.4 and 0.7 ± 0.3 log CFU mL^−1^, respectively) (*P* ≤ 0.05). Counts of *S. enterica* in water after WUV + PA treatment and NaClO sanitization were not statistically different, in which populations were below the detection limit (<50 CFU mL^−1^). Undoubtedly, bacterial cells that are washed off from the fruit product were inactivated by the UV‐C and/or the sanitizer present in the washing solution, thereby reducing the risk of potential cross‐contamination. Regarding *L. monocytogenes* survival after washing with chemicals in washing water, the use of chlorine sanitization was significantly better than the combined treatment disinfection (*P* ≤ 0.05, Table 5). Even when WUV + PA combinations did not enhance the inactivation of inoculated pathogens in water wash compared to chlorine disinfection, the combined non‐toxic chemical–physical treatments are still recommendable due to their increased effectiveness at decontaminating the food matrix, leaving the wash water free of potential mutagenic and carcinogenic products. Indeed, UV‐C light has been used widely as a non‐thermal method of disinfecting drinking and wastewater.^40^ The amount of wastewater generated per mass unit of product also depends on the disinfection technique employed, UV‐C light being capable of disinfecting efficiently both the process water and the product.^41^

**Table 5:** Mean and standard deviation (SD) values (n = 3) of the population of (A) *Salmonella enterica* or (B) *Listeria monocytogenes* in washing water after 2 min of disinfection treatments

Disinfection treatment^a^	Population (log CFU mL^−1^) (mean ± SD)^b^	
	*L. monocytogenes*	*S. enterica*
H_2_O	4.50 ± 0.14^Aa^	4.57 ± 0.27^Aa^
NaClO	0.1 ± 0.2^Ac^	0.0 ± 0.0^Ac^
WUV	0.87 ± 0.42^Ab^	0.73 ± 0.55^Ab^
WUV + PA	0.49 ± 0.33^Ab^	0.13 ± 0.25^Bc^

^a^ H_2_O: control samples (washing with tap water), NaClO: hypochlorite solution (200 mg L^−1^), WUV: ultraviolet disinfection, WUV + PA: ultraviolet disinfection combined with peracetic acid (40 mg L^−1^).

^b^ Upper case letters indicate significant differences between pathogenic bacteria according to the Student t‐test (*P* < 0.05). Lower case letters indicate significant differences among disinfection treatments according to the Tukey HSD *post hoc* test (*P* < 0.05).

### Effect of the storage conditions (time and temperature) and food matrix on the survival of pathogenic bacteria on strawberries

The effects of the storage conditions on both pathogenic bacteria on whole and fresh‐cut strawberries after 7‐days at both refrigeration temperatures (4 and 10 °C) are presented in Table 6. Considering all variables, the population of *L. monocytogenes* decreased at the end of the experimental period, with reductions ranging from 0.3 to 3.2 log_stor_. In the case of *S. enterica*, microbial reductions during storage ranged between 0.6 to 1.9 log_stor_. These results are in agreement with previous publications,^7^, ^8^, ^42^ reporting that populations of *Salmonella* spp. and *L. monocytogenes* decreased by 1.0–2.0 logs on the surface of strawberries stored at refrigeration temperatures (4 and 10 °C).

**Table 6:** Mean and standard deviation (SD) values (n = 3) of the log reduction (Log_stor_) of *S. enterica* and *L. monocytogenes* as a function of the storage conditions after disinfection

*L. monocytogenes*				*S. enterica*			
Matrix	Disinfection treatment^a^	Temperature ( °C)	Log_stor_ (mean ± SD)^b^	Matrix	Disinfection treatment^a^	Temperature ( °C)	Log_stor_ (mean ± SD)^b^
Whole	H_2_O	10	1.12 ± 0.82^*Aa^	Whole	H_2_O	10	1.01 ± 0.87*^Aab^
	NaClO	10	1.54 ± 0.38^*Aa^		NaClO	10	1.26 ± 0.32*^Abc^
	WUV	10°	0.57 ± 1.40^*Aa^		WUV	10	0.55 ± 0.28*^Aa^
	WUV + PA	10	0.55 ± 0.48^*Aa^		WUV + PA	10	1.84 ± 0.34*^Ac^
Fresh cut	H_2_O	10	2.90 ± 1.60^*Aa^	Fresh cut	H_2_O	10	0.68 ± 0.17*^Aa^
	NaClO	10	1.71 ± 1.65^*Aa^		NaClO	10	1.04 ± 0.22*^Aa^
	WUV	10	3.23 ± 1.16^*Aa^		WUV	10	1.59 ± 0.55*^Ba^
	WUV + PA	10	0.26 ± 1.52^*Aa^		WUV + PA	10	1.91 ± 0.53*^Aa^
Whole	H_2_O	4	1.46 ± 1.32^*Aa^	Whole	H_2_O	4	1.91 ± 1.57*^Aa^
	NaClO	4	1.53 ± 0.36^*Aa^		NaClO	4	1.33 ± 0.27*^Aa^
	WUV	4	0.98 ± 0.97^*Aa^		WUV	4	0.75 ± 0.31*^Aa^
	WUV + PA	4	0.61 ± 0.46^*Aa^		WUV + PA	4	1.20 ± 0.22** ^Aa^
Fresh cut	H_2_O	4	2.51 ± 0.21^*Aa^	Fresh cut	H_2_O	4	1.16 ± 0.22*^Ba^
	NaClO	4	1.35 ± 2.26^*Aa^		NaClO	4	1.38 ± 0.15*^Aa^
	WUV	4	3.19 ± 0.39^*Aa^		WUV	4	1.41 ± 0.09*^Ba^
	WUV + PA	4	0.74 ± 0.69^*Aa^		WUV + PA	4	1.33 ± 1.16** ^Aa^

^a^ H_2_O: control samples (washing with tap water), NaClO: hypochlorite solution (200 mg L^−1^), WUV: ultraviolet disinfection, WUV + PA: ultraviolet disinfection combined with peracetic acid (40 mg L^−1^).

^b^ Upper case letters indicate significant differences between pathogenic bacteria according to the Student *t‐*test (*P* < 0.05). Lower case letters indicate significant differences among disinfection treatments according to the Tukey HSD *post hoc* test (*P* < 0.05).

** Asterisks indicate significant differences between storage temperatures according to the Student *t‐*test (*P* < 0.05).

According to the model estimates, the UV‐C (single and combined with PA) treatments had a larger impact on the inactivation kinetics of *S. enterica* than the other sanitizing treatments had, decreasing significantly microbial concentration after storage (*P* ≤ 0.05) (Table 7). However, the WUV + PA treatment decreased *L. monocytogenes* population until the end of storage but in a lesser extent compared to the inactivation of *S. enterica. Listeria monocytogenes* decreased equally with no statistical difference between UV‐C and chlorine sanitization (*P* > 0.05) (Table 8).

**Table 7:** Estimations of the generalized linear regression models for the calculated log reductions after a 7 day storage (Log_stor_) of *S. enterica* in whole and fresh‐cut strawberries

Variable^**a**^	Coefficient (95%, C.I.)	S.E.	*P*‐value^b^
Intercept	0.60 (−0.07, 1.27)	0.33	0.081
NaClO	0.53 (−0.31, 1.38)	0.42	0.208
*WUV*	*0.92 (0.07, 1.76)*	*0.42*	*0.035*
*WUV + PA*	*1.35 (0.51, 2.20)*	*0.42*	*0.003*
Whole	0.50 (−0.31, 1.33)	0.40	0.222
Temp 4° C	0.64 (−0.19, 1.46)	0.40	0.124
NaClO * Whole^c^	−0.47 (−1.45, 0.51)	0.48	0.337
WUV * Whole	*−1.40 (−2.38, −0.42)*	*0.48*	*0.006*
WUV + PA * Whole	−0.65 (−1.63, 0.33)	0.48	0.185
NaClO * Temp 4° C	−0.48 (−1.46, 0.50)	0.48	0.325
WUV * Temp 4° C	−0.67 (−1.65, 0.30)	0.48	0.170
WUV + PA * Temp 4° C	*−1.29 (−2.27, −0.32)*	*0.48*	*0.011*
Whole * Temp 4° C	0.09 (−0.55, 0.74)	0.32	0.772
**Goodness of fit statistics** ^d^	RMSE: 0.725	AIC: 80.83	BIC: 105.80

^a^ Reference categories in the models were: disinfection treatment = H_2_O (control); Matrix = fresh‐cut strawberry; and Temp = 10 °C.

^b^ Variables considered significant (*P* < 0.05) are in italics.

^c^ Interaction terms.

^d^ RMSE, root mean squared error; AIC, Akaike information criterion; BIC, Bayesian information criterion.

**Table 8:** Estimations of the generalized linear regression models for the calculated log reductions after a 7 day storage (Log_stor_) of *Listeria monocytogenes* in whole and fresh‐cut strawberries

Variable^**a**^	Coefficient (95%, C.I.)	S.E.	*P*‐value^b^
Intercept	*2.79 (1.62, 3.95)*	*0.57*	*<0.001*
NaClO	−1.10 (−2.67, 0.48)	0.78	0.166
WUV	0.40 (−1.18, 1.97)	0.78	0.611
*WUV + PA*	*−2.36 (−3.93, −0.78)*	*0.78*	*0.004*
Whole	*−1.56 (−2.99, −0.12)*	*0.71*	*0.035*
Temp 4 °C	−0.16 (−1.60, 1.27)	0.71	0.818
NaClO * Whole^c^	1.43 (−0.39, 3.24)	0.90	0.121
WUV * Whole	−1.02 (−2.84, 0.80)	0.90	0.265
WUV + PA * Whole	1.50 (−0.32, 3.32)	0.90	0.104
NaClO * Temp 4 °C	−0.16 (1.98, 1.66)	0.90	0.859
WUV * Temp 4 °C	0.21 (−1.61, 2.03)	0.90	0.819
WUV + PA * Temp 4 °C	0.30 (−1.52, 2.12)	0.90	0.741
Whole * Temp 4 °C	0.28 (−1.00, 1.56)	0.63	0.664
**Goodness of fit statistics** ^d^	RMSE: 1.047	AIC: 157.99	BIC: 184.18

^a^ Reference categories in the models were: disinfection treatment = H_2_O (control); Matrix = fresh‐cut strawberry; and temperature = 10 °C.

^b^ Variables considered significant (*P* < 0.05) are in italics.

^c^ Interaction terms.

^d^ RMSE, root mean squared error; AIC, Akaike information criterion; BIC, Bayesian information criterion.

Regarding the effect of storage temperature (4 or 10 °C) after 7‐days, the behavior of *L. monocytogenes* and *S. enterica* was similar at both refrigeration temperatures studied with no significant differences observed between bacteria (*P* > 0.05). However, the interaction of WUV + PA × 4 °C (treatment × temperature) for *S. enterica* was significantly associated with an increased risk, as the reductions (log_stor_) in the pathogenic bacteria were lower than the samples disinfected with the WUV + PA treatment and stored at 10 °C (Tables 7 and 8).

Regarding the strawberry matrix (whole and fresh cut), the results showed that *L. monocytogenes* concentration on whole samples remained higher than the fresh‐cut samples throughout the experimental period (Table 8). For *S. enterica*, no significant differences between matrices were reported, either for temperature or for the treatments applied, apart from the single interaction term of WUV × whole (treatment × matrix) (Table 7). These results contrast with those obtained by previous investigations, which reported a 3.0‐log reduction of *L. monocytogenes* after 7 day storage in intact strawberries, while the populations on cut surfaces remained constant.^7^ Populations of *Salmonella* spp. also decreased by 1.0 log on the surfaces of intact strawberries but remained constant in fresh‐cut fruits at 5 °C.^8^ These studies also demonstrated that the soluble solid content and humidity on cut surfaces could provide adequate nutrition to enhance the bacterial survival on fresh‐cut samples. In our case, the inoculation of the pathogen for both whole and fresh‐cut strawberries was done on the surface of the strawberry, being the conditions similar. Beside the storage conditions and variables studied, therefore, the efficacy of different decontamination treatments on bacteria survival depends largely on the microorganisms present in the food and on the strains variability.^43^ The sublethal damage that disinfection treatments may have caused to microorganisms is also an important factor to consider, as stress resistance also varies between strains or serovars of the same species.^44^ Variability therefore plays a large role in the efficacy of a decontamination treatment and bacterial survival and should be considered in microbial risk assessments and shelf‐life estimations.^45^

### Effect of the sanitizing treatments and experimental conditions on infectivity of MNV‐1 on strawberries

The initial virus load was 3.1 ± 0.5 log TCID_50_ mL^−1^ on artificially inoculated strawberries. The reductions in virus titer after disinfection treatments ranged from 1.3 to 1.7 log_dis_ (Table 9). Inactivation of MNV‐1 was significantly lower on strawberries treated with the one‐sided DUV irradiation without immersion, reducing viable infectivity by ca. 1.4 log_dis_. These results agreed with previous studies.^18^ In our study, the shading effect could be negligible as the inoculated surface of strawberries was exposed to DUV light. However, when reaching industrial levels, particular attention must be paid to this factor, to expose all strawberry surface to UV‐C light. In this regard, WUV method represents an advantage as equal irradiation to all sites of strawberries is facilitated by water agitation. Butot *et al*. (2018)^34^ reported similar results with reductions about <2 log (TCID_50_ g^−1^) of MNV‐1 on fresh and frozen strawberries exposed to DUV for 20 s (average fluence of 2.1 ± 0.3 kJ m^−2^), 60 s (average fluence of 6.5 ± 0.7 kJ m^−2^) and 120 s (average fluence of 13.3 ± 1.0 kJ m^−2^). On the other hand, WUV without chemical agents did not achieve effective inactivation compared with WUV + PA and NaClO sanitation (*P* ≤ 0.05). Thus, sanitizing agents should be used to enhance the antiviral effect on the disinfection of strawberries. Moreover, PA is considered as an important alternative to chlorine for washing produce; however, there is limited information regarding its efficacy against foodborne enteric viruses.^6^ The efficacy of chemical washing treatments was therefore significantly higher for the inhibition of MNV‐1 infectivity on strawberries (*P* ≤ 0.05) after washing procedures, demonstrating that there was a physical removal of the enteric viruses during washing sanitation combined with the viricidal effect of the sanitizing agent, with reductions about ca. 2 log for WUV + PA and NaClO. Previous investigations reported that in the UV‐PA combination, there PA photolysis occurs under the action of the UV light. According to Caretti and Lubello *et al*. (2003)^46^ there is an disruption in the O–O bond of the PA molecule, with the subsequent formation of the hydroxyl radical.

**Table 9:** Mean and standard deviation (SD) values (n = 3) of the log reduction (Log_dis_) of murine norovirus (MNV‐1) infectivity as a function of the applied disinfection treatments

Disinfection treatment^1^	Log_dis_ (mean ± SD)^2^	
DUV	1.41 ± 0.11^c^	
NaClO	1.70 ± 0.13^a^	
WUV	1.33 ± 0.06^c^	
WUV + PA	1.58 ± 0.06^b^	

^1^ DUV: conventional dry UV‐C light, NaClO: hypochlorite solution (200 mg L^−1^), WUV: ultraviolet disinfection, WUV + PA: ultraviolet disinfection combined with peracetic acid (40 mg L^−1^).

^2^ Lowercase letters indicate significant differences between disinfection treatments according to the Tukey HSD *post hoc* test (*P* < 0.05).

The use of the different treatments tested in the present study together with the storage variables (time and temperature) and food matrix (whole and fresh‐cut) demonstrated a slightly impact on virus inactivation during the whole storage period (7d) (Table 10). Thus, the viability MNV‐1 infectivity declined between 0.0 and 0.3 log_stor_ until the end of the experiment, regardless of temperature, treatment, and matrix (*P* > 0.05). Indeed, the effect of both refrigerated storage temperature (4 °C and 10 °C) has not been significant for the infectivity of MNV‐1 in the present study. It is known that viruses can survive in foods with high stability for many days to weeks, surviving at fresh and frozen storage conditions.^47^, ^48^, ^49^, ^50^, ^51^ Verhaelen *et al*. (2012)^10^ demonstrated that murine norovirus decayed rapidly on strawberries with TFL values (time for the first log‐unit reduction) of only 1 day (95% CI of 0.6–1 days), but persisted to the end of the shelf life of strawberries at 4 °C and 10 °C. Similarly, Mattison *et al*. (2007)^52^ studied the persistence of feline calicivirus (FCV), a human norovirus surrogate used before the discovery of MNV‐1, on strawberries. They showed that also FCV is reduced rapidly on strawberries with a sharp initial decrease at room temperature, which was not found if strawberries were stored at 4 °C. The longer survival times at lower temperatures shown by our data are consistent with previous studies. Lee *et al*. (2015)^53^ reported that hepatitis A virus (HAV), MNV‐1, and bacteriphage MS2 on peppers were observed to remain infectious until 14 days after inoculation at 4 °C. It is not possible to make a direct comparison with previous studies due to differences in experimental conditions but the results are similar, demonstrating that lower temperature conditions decreased the reduction of viruses on foods. Considering that vegetables and fruits are typically stored under refrigerated conditions, the ability of foodborne viruses to survive at 4 °C should be taken into consideration.

**Table 10:** Mean and standard deviation (SD) values (n = 3) of the log_10_ reduction (Log_stor_) of murine norovirus (MNV‐1) infectivity as a function of the storage conditions after disinfection

Matrix	Disinfection treatment^a^	Temperature ( °C)	Log_stor_ (mean ± SD)^b^
Whole	DUV	10	−0.08 ± 0.14^Ab^ *
	NaClO	10	0.13 ± 0.22^Aab^ *
	WUV	10	0.29 ± 0.07^Aa^ *
	WUV + PA	10	0.17 ± 0.19^Aa^ *
Fresh cut	DUV	10	−0.08 ± 0.14^Ab^ *
	NaClO	10	0.13 ± 0.13^Aab^ *
	WUV	10	0.17 ± 0.14^Aa^ *
	WUV + PA	10	0.17 ± 0.14^Aa^ *
Whole	DUV	4	0.04 ± 0.14^Aa^ *
	NaClO	4	0.00 ± 0.21^Aa^ *
	WUV	4	0.17 ± 0.07^Aa^ *
	WUV + PA	4	0.13 ± 0.13^Aa^ *
Fresh cut	DUV	4	0.04 ± 0.07^Aa^ *
	NaClO	4	0.08 ± 0.19^Aa^ *
	WUV	4	0.17 ± 0.14^Aa^ *
	WUV + PA	4	0.13 ± 0.13^Aa^ *

^a^ DUV: conventional dry UV‐C light, NaClO: hypochlorite solution (200 mg L^−1^), WUV: ultraviolet disinfection, WUV + PA: ultraviolet disinfection combined with peracetic acid (40 mg L^−1^).

^b^ Upper case letters indicate significant differences between matrices according to the Student *t‐*test (*P* < 0.05). Lower case letters indicate significant differences between disinfection treatments according to the Tukey HSD *post hoc* test (*P* < 0.05).

* Asterisks indicate significant differences between storage temperatures according to the Student *t‐*test (*P* < 0.05).

Likewise, no differences were observed in the present study regarding the infective ability of MNV‐1 on whole or fresh‐cut strawberries, probably due to the fact that the inoculation was made on the surface of the strawberry in both cases. Results and previous investigations illustrate the importance of using data obtained with food matrices and the storage temperature of interest when developing food safety strategies related to foodborne enteric viruses.

## Conclusion

Currently, little information on the behavior of *S. enterica, L. monocytogenes* and MNV‐1 in whole and fresh‐cut strawberries during their entire shelf‐life has been available. The results obtained in the present study reported that the combined treatment of UV‐C at 40 mg L^−1^ PA dose showed a significant effect on the reduction of the pathogens in comparison to the individual water‐assisted UV‐C control treatment in the evaluated food matrices, and that this treatment can therefore be recommended as its efficacy was equivalent to chlorine sanitization. Although disinfection treatment operations are capable of reducing the incidence of pathogenic microorganisms in strawberries, operating conditions and hygiene practices during storage, commercialization, and consumption will define the fate of pathogens and microbial risk to consumers. In fact, MNV‐1 infectivity is able to persist on strawberries stored at refrigeration temperatures. Data from this study therefore indicate the necessity of considering several factors when and if microbiological limits are established for the pathogenic microorganisms selected on whole and fresh‐cut strawberries during storage and shelf life.
